# Relationship between serum iohexol clearance, serum SDMA concentration, and serum creatinine concentration in non‐azotemic dogs

**DOI:** 10.1111/jvim.15659

**Published:** 2019-11-14

**Authors:** Myles McKenna, Ludovic Pelligand, Jonathan Elliott, Daniel Cotter, Rosanne Jepson

**Affiliations:** ^1^ Department of Clinical Science and Services Royal Veterinary College London United Kingdom; ^2^ Department of Comparative Biomedical Sciences Royal Veterinary College London United Kingdom

**Keywords:** canine, diagnosis, glomerular filtration rate, renal

## Abstract

**Background:**

Serum creatinine and symmetric dimethylarginine (SDMA) are used as surrogate markers of glomerular filtration rate (GFR) in clinical practice. Data pertaining to the correlations between GFR, SDMA, and serum creatinine in client‐owned dogs are limited.

**Objectives:**

To describe the relationship between GFR, SDMA, and serum creatinine in a population of client‐owned dogs, and to compare clinical utility of SDMA to GFR estimation for detecting pre‐azotemic chronic kidney disease.

**Animals:**

Medical records of 119 dogs that had GFR estimation performed via serum iohexol clearance between 2012 and 2017.

**Methods:**

Prospective study using archived samples. GFR, SDMA, and serum creatinine results were reviewed and submitting practices contacted for outcome data. All dogs included in the study population were non‐azotemic. Correlations between GFR, SDMA, and serum creatinine were determined by regression analysis. Sensitivity, specificity, and positive and negative likelihood ratios of different cutoffs for SDMA and serum creatinine for detecting decreased GFR were calculated, using a 95% confidence interval.

**Results:**

Serum creatinine and SDMA were moderately correlated with GFR (R^2^ = 0.52 and 0.27, respectively, *P* < .0001) and with each other (R^2^ = 0.33, *P* < .0001). SDMA >14 μg/dL was sensitive (90%) but nonspecific (50%) for detecting a ≥40% decrease in GFR. Optimal SDMA concentration cutoff for detecting a ≥40% GFR decrease was >18 μg/dL (sensitivity 90%, specificity 83%).

**Conclusions and Clinical Importance:**

In non‐azotemic dogs being screened for decreased renal function, using a cutoff of >18 μg/dL rather than >14 μg/dL increases the specificity of SDMA, without compromising sensitivity.

**Abbreviations**
CI: confidence interval
CKD: chronic kidney disease
GFR: glomerular filtration rate
HPCE: high‐performance capillary electrophoresis
LR+: positive likelihood ratio
LR−: negative likelihood ratio
ROC: receiver operating characteristic
RVC: Royal Veterinary College
SDMA: symmetric dimethylarginine

## 1 INTRODUCTION

Glomerular filtration rate (GFR) estimation is regarded as the gold standard method for assessing renal function, as it is directly proportional to renal mass.^1^ Although direct measurement of GFR is not possible, it can be estimated by assessing the clearance of a marker of GFR.^2^ Measuring the plasma clearance of iohexol has become a widely used means of estimating GFR due to its availability, cost, and ease of use.^3^, ^4^, ^5^, ^6^, ^7^, ^8^, ^9^, ^10^, ^11^, ^12^ Measuring the plasma clearance of iohexol using a limited sampling technique has been described in dogs.^13^

Although GFR estimation is the gold standard for assessing renal function, measurement of serum creatinine, a surrogate marker of GFR, remains the main means of assessing renal function in dogs in clinical practice.^14^ However, using serum creatinine as a marker of GFR has limitations. The relationship between serum creatinine and GFR is exponential, such that serum creatinine has limited sensitivity for the early detection of declining renal function.^15^ In addition, lean body mass has an effect on serum creatinine concentrations,^16^ making assessment of GFR in well‐muscled or cachexic animals challenging. Furthermore, false increases in serum creatinine concentrations are possible with certain assays,^17^ and in male dogs, a small amount of creatinine is secreted in the renal tubules.^18^

Recent studies indicate that symmetric dimethylarginine (SDMA) could be a promising marker of GFR in dogs.^19^, ^20^ Symmetric dimethylarginine is produced by the breakdown of proteins, the arginine residues of which have been posttranslationally methylated and is excreted primarily (≥90%) by renal clearance.^21^, ^22^ Unlike serum creatinine, SDMA is unaffected by lean body mass.^16^ Symmetric dimethylarginine has an exponential relationship with GFR but could be a more sensitive marker of declining GFR than serum creatinine.^19^ A caveat is that data pertaining to the effects of concurrent disease on SDMA remain somewhat limited.

In dogs with X‐linked hereditary nephropathy, SDMA increases as the disease progresses, correlating with increases in serum creatinine and decreasing GFR.^19^ In this population of dogs, SDMA detects, on average, a <20% decrease in GFR‐ earlier than serum creatinine using any comparison method.^19^ In addition, serum SDMA increases before serum creatinine by a mean of 9.8 months (range, 2.2‐27 months) in dogs with chronic kidney disease (CKD).^20^ Although the 2 aforementioned studies described the relationship between GFR, SDMA, and serum creatinine, the study populations in both cases were relatively small populations of research colony dogs. Data relating to the relationship between GFR, SDMA, and serum creatinine in a general population of client‐owned dogs in a clinical setting where kidney disease is suspected based on their clinical presentation are lacking.

The aim of this study was to describe the relationship between GFR, SDMA, and serum creatinine in a population of client‐owned dogs presenting to both referral and first‐opinion practice and to compare the clinical utility of SDMA to the gold standard of GFR estimation via serum iohexol clearance for the detection of pre‐azotemic CKD.

## 2 MATERIALS AND METHODS

### 2.1 Data acquisition and analysis

The medical records of dogs that had samples submitted for GFR estimation via iohexol clearance to the Royal Veterinary College (RVC) from September 3, 2012, to April 11, 2017, were assessed. The project was reviewed and approved by the RVC clinical research and ethical review board which allowed for access to joint iohexol clearance test submission forms held by deltaDOT Ltd. and the RVC, with subsequent submission of serum samples for SDMA and serum creatinine measurement via batch analysis to Idexx Laboratories Ltd. Additionally, contact with the veterinarians for access to the clinical records of the dogs under investigation and for completion of a short questionnaire regarding case outcomes was approved. This contact was performed before final implementation of the General Data Proection Regulation (EU) 2016/679.

### 2.2 Iohexol clearance protocol

A standard protocol was used for performing the iohexol clearance tests via a limited sampling technique.^23^ A single dose of iohexol (Omnipaque™ 300) was administered at 300 mg iodine/kg intravenously through an IV catheter. Blood was collected at precisely 2, 3, and 4 hours after iohexol administration, and samples were subsequently centrifuged. Serum iohexol concentration for each serum sample was measured using deltaDOT Ltd.'s previously validated high‐performance capillary electrophoresis method.^24^ Glomerular filtration rate was estimated from the iohexol clearance data by application of a compartmental model and a dog‐specific correction formula,^13^ normalized to body weight in kilograms. For data analysis, dogs were divided into the 4 weight quartiles^13^: Category 1:1.8‐12.4 kg, Category 2:13.2‐25.5 kg, Category 3:25.7‐31.6 kg, and Category 4:32.0‐70.3 kg. In the event that a canine body weight did not fall within the range of 1 of these body weight categories, the dog was included in the body weight category to which its body weight was closest. The estimated GFR of each dog was compared to the mean GFR of their respective body weight categories^13^: 2.89 mL/kg/min for Category 1, 2.4 mL/kg/min for Category 2, 2.16 mL/kg/min for Category 3, and 2.25 mL/kg/min for Category 4. Glomerular filtration rate estimation results were interpreted in light of the following categorization criteria:^23^ GFR group 1: GFR increased or <20% decreased from the mean GFR of the body weight category, kidney disease considered excluded or very unlikely as an etiology for presenting clinical signs; GFR group 2: ≥20% but <30% decrease in GFR from the mean GFR of the body weight category, kidney disease considered possible but unconfirmed as an etiology for presenting clinical signs; GFR group 3: ≥30% but <40% decrease in GFR from the mean GFR of the body weight category, kidney disease considered likely as an etiology for presenting clinical signs; GFR group 4: ≥40% decrease in GFR from the mean GFR of the body weight category, kidney disease considered almost certain as an etiology for presenting clinical signs.

Using the baseline serum sample that was submitted for GFR estimation via iohexol clearance testing, SDMA and serum creatinine concentrations were measured. The serum used was collected before iohexol administration in all cases. After storage in a −80°C freezer for a median of 166 days (range, 9‐1360 days), all samples were submitted to Idexx Laboratories Ltd. All serum creatinine samples were sent to Idexx Wetherby (UK; analyzer: Olympus AU5800, method: Jaffe kinetic without deproteination). Symmetric dimethylarginine samples were sent to either Idexx Wetherby (UK) or Idexx Ludwigsburg (Germany). Identical analyzers and methodology were used to measure SDMA in both laboratories (analyzer: Olympus AU5800, method: enzyme immunoassay). The reference intervals for SDMA and serum creatinine used in this study were set at 0‐14 μg/dL and 0.23‐1.63 mg/dL, respectively, on September 29, 2017. Based on the Idexx reference interval for serum creatinine, only data from dogs that were non‐azotemic were taken forward for further analysis.

Reasons for performing GFR estimation in this population are described in a separate publication evaluating the clinical utility of GFR estimation in dogs,^23^ and included screening for pre‐azotemic CKD, confirmation of azotemic CKD, screening for pre‐azotemic acute kidney injury, and assessing the need for carboplatin dose adjustment in patients undergoing chemotherapy. As part of that study, the veterinarian(s) who submitted each sample set for iohexol clearance to be measured were contacted via email and asked to complete a short questionnaire regarding case outcomes. Data collected included status (ie, alive/dead), date of euthanasia/death, reason for euthanasia/death if known, and whether or not a diagnosis was reached for the clinical signs/ routine laboratory findings that prompted GFR estimation. If no response to the questionnaire was received or the answers were insufficient to provide outcome information, veterinarians were contacted directly by telephone to request the full clinical history and laboratory reports for the dogs in question from the time of iohexol clearance sample submission to the time of follow‐up.

### 2.3 Statistical analysis

Data are presented as median (range) unless otherwise stated. GFR estimation results were expressed as percent deviation from the mean GFR of that canine body weight category^13^ and interpreted in light of the defined categorization criteria.^23^ Statistical analyses were performed using statistical software Prism 8 (Prism 6 for Mac OS X, GraphPad Software Inc, La Jolla, California). To investigate the associations between SDMA and GFR, serum creatinine concentration and GFR, and SDMA and serum creatinine concentration, SDMA and serum creatinine results were plotted against GFR data and against each other. Best‐fit equations were derived from the resulting data plots in order to measure the associations between variables. Data from the 119 dogs for which GFR results and creatinine and/or SDMA results were available were included in the calculation of these associations.

The sensitivity, specificity, positive likelihood ratio (LR+) and negative likelihood ratio (LR−) of different cutoff points for SDMA (>10, >12, >14, >16, >18, and >20 μg/dL) and different cutoff points for serum creatinine (≥1.0, ≥1.1, ≥1.2, ≥1.3, ≥1.4, and ≥1.5 mg/dL) for detecting decreases in GFR (GFR decreases of ≥20%, ≥30%, and ≥ 40% below the mean GFR of the patient's body weight category, respectively) were manually calculated, using a 95% confidence interval (CI). For calculation of sensitivity, specificity, LR+, and LR−, only those dogs for which GFR, SDMA, and serum creatinine results were simultaneously available were included (n = 90).

Receiver operating characteristic (ROC) curves were developed to assess the trade‐off between the sensitivity and (1 − specificity) across a series of cutoff points for SDMA (>10, >12, >14, >16, >18, and >20 μg/dL) and serum creatinine (≥1.0, ≥1.1, ≥1.2, ≥1.3, ≥1.4, and ≥ 1.5 mg/dL) to detect decreases in GFR of ≥20%, ≥30% and ≥40% below the mean GFR of a patient's body weight category. Based off ROC curve data, Youden's J statistic was used to determine optimal cutoff points.

## 3 RESULTS

### 3.1 GFR, serum creatinine, and SDMA results

A total of 132 dogs had samples submitted for GFR estimation between September 3, 2012, and April 11, 2017. The characteristics of this study population are described.^23^ Serum creatinine data were available for 100 of 132 (76%) dogs. In 4 of the 100 (4%) dogs for which serum creatinine was available, serum creatinine was above the upper limit of the reference interval (>1.63 mg/dL). The 4 azotemic dogs were excluded from the data set and from further analysis, leaving a total of 128 dogs in the data set. After exclusion of the 4 azotemic dogs, serum creatinine data were available for 96 of 128 (75%) dogs. Symmetric dimethylarginine was available for 113 of 128 (88%) dogs with concurrent SDMA and creatinine available for 90 (70%) dogs.

In the 119 dogs for which either serum creatinine or SDMA was available, median GFR was 2.19 mL/kg/min across all body weight categories with a range of 1.16‐4.04 mL/kg/min. Percentage deviation from the mean of the body weight category ranged from −55% to +68.0% with a median deviation of −11.2% from the mean. In 42 of 119 (35%) dogs, the percentage deviation from the mean of the body weight category was ≥20% decreased.

Median serum creatinine for all 96 non‐azotemic dogs for which it was available was 1.11 mg/dL (range, 0.45‐1.59 mg/dL), whereas median SDMA for all 113 dogs for which it was available was 15 μg/dL (range, 6‐27 μg/dL).

In 59 of 113 (52%) dogs, SDMA was above the upper limits of the reference interval (>14 μg/dL). The median SDMA for these 59 dogs was 17 μg/dL (range, 15‐27 μg/dL). Median change in GFR from the mean GFR for the body weight category in the 59 dogs with increased SMDA was −18.8% (range, −55% to +40.8%). In the 59 cases with increased SDMA, GFR was increased or <20% decreased below the mean GFR for the canine body weight category in 32 of 59 (54%) of cases. Further description of SDMA and serum creatinine results by GFR category is provided in Table 1.

**Table 1:** Number of patients falling into each GFR category with normal (≤14 μg/dL) or increased (>14 μg/dL) SDMA, and serum creatinine concentrations above and below the cutoff for stage 1 chronic kidney disease (≥1.4 mg/dL), as per the International Renal Interest Society (IRIS) guidelines^25^

Variables	All GFR categories (total)	GFR ↑ or ↓ <20%	GFR ↓ ≥20 but <30%	GFR ↓ ≥30 but <40%	GFR ↓ ≥40
SDMA ≤14 μg/dL					
Number	54	43 (80%)	3 (5.5%)	5 (9%)	3 (5.5%)
Median SDMA μg/dL (range)	11 (6‐14)	11 (6‐14)	11 (10‐12)	13 (11‐14)	12 (12‐14)
SDMA >14 μg/dL					
Number	59	32 (54.0%)	10 (17.0%)	5 (8.5%)	12 (20.5%)
Median SDMA μg/dL (range)	17 (15‐27)	16 (15‐24)	18 (15‐22)	21 (16‐25)	21 (16‐27)
Creatinine <1.4 mg/dL					
Number	82	59 (72%)	11 (13%)	8 (10%)	4 (5%)
Median creatinine mg/dL (range)	1.05 (0.45‐1.39)	0.96 (0.45‐1.37)	1.19 (0.80‐1.39)	1.33 (0.90‐1.39)	1.23 (0.87‐1.38)
Creatinine ≥1.4 mg/dL					
Number	14	2 (14.3%)	3 (21.4%)	2 (14.3%)	7 (50.0%)
Median creatinine mg/dL (range)	1.46 (1.40‐1.59)	1.45 (1.44‐1.45)	1.58 (1.55‐1.59)	1.47 (1.46‐1.48)	1.46 (1.40‐1.50)

*Note*: Glomerular filtration rate results are expressed as per the categories defined by McKenna et al^23^: GFR group 1: GFR increased or <20% decreased from the mean GFR of the body weight category, kidney disease considered excluded/or very unlikely; GFR group 2: ≥20% but <30% decrease in GFR, kidney disease considered possible but unconfirmed; GFR group 3: ≥30% but <40% decrease in GFR, kidney disease considered likely; GFR group 4: ≥40% decrease in GFR, kidney disease considered almost certain.

Abbreviations: GFR, glomerular filtration rate; SDMA, symmetric dimethylarginine.

### 3.2 The relationship between SDMA and serum creatinine concentration with GFR

**Figure 1:**
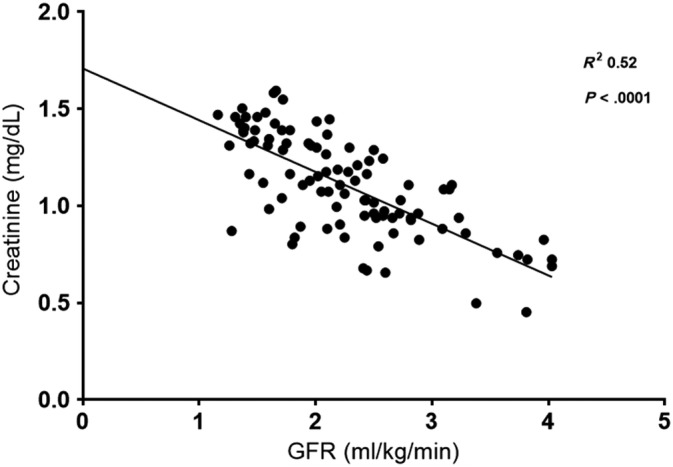
Relationship between serum iohexol clearance and serum creatinine. GFR, glomerular filtration rate

**Figure 2:**
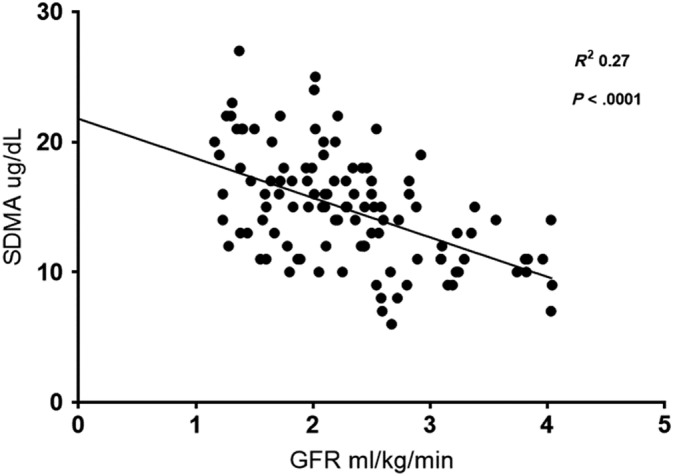
Relationship between serum iohexol clearance and serum symmetric dimethylarginine (SDMA)

**Figure 3:**
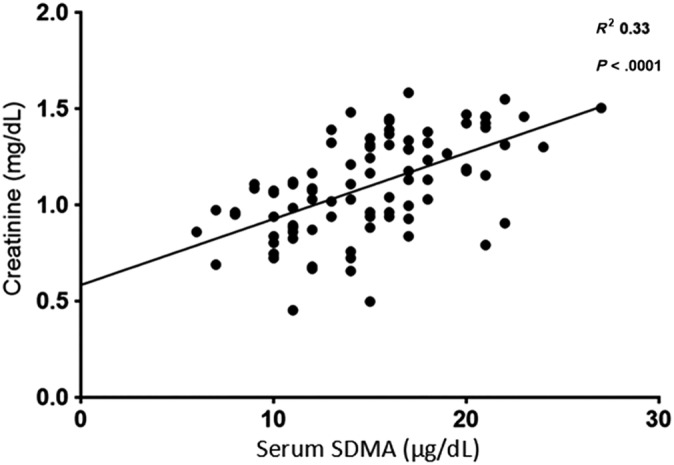
Relationship between serum symmetric dimethylarginine (SDMA) and serum creatinine

Serum creatinine and SDMA were moderately correlated with GFR (*R* ^2^ = 0.52 and 0.27 respectively, *P* < .0001) and with each other (*R* ^2^ = 0.33, *P* < .0001). The relationships between GFR and serum creatinine (Figure 1), GFR and SDMA (Figure 2), and serum creatinine and SDMA (Figure 3) were all linear.

### 3.3 Utility of SDMA for detecting decreases in GFR

The manually calculated sensitivity and specificity of different cutoff points for SDMA (>10, >12, >14, >16, >18, and >20 μg/dL) and different cutoff points for serum creatinine (≥1.0, ≥1.1, ≥1.2, ≥1.3, ≥1.4, and ≥ 1.5 mg/dL) for detecting decreases in GFR (GFR decreases of ≥20%, ≥30% and ≥ 40% below the mean GFR of the canine body weight category respectively) are outlined in Table 2. The LR+ and LR− of these cutoff points for SDMA and serum creatinine for detecting decreases in GFR were manually calculated according to the following equations and are outlined in Table 3.

**Table 2:** Ability of different cutoff points of SDMA and serum creatinine to detect different categories of %GFR decrease below the mean GFR of the dog's body weight category

Variables	Sample size	Sensitivity %GFR ↓ ≥20% (95% CI)	Specificity %GFR ↓ ≥20% (95% CI)	Sensitivity %GFR ↓ ≥30% (95% CI)	Specificity %GFR ↓ ≥30% (95% CI)	Sensitivity %GFR ↓ ≥40% (95% CI)	Specificity %GFR ↓ ≥40% (95% CI)
SDMA > 10 μg/dL	90	96.8 (83.3‐99.9)	22.0 (12.3‐34.7)	94.7 (74.0‐99.9)	18.3 (10.1‐29.3)	90.9 (58.7‐99.8)	16.5 (9.1‐26.5)
SDMA > 12 μg/dL	90	83.9 (66.3‐94.6)	42.4 (29.6‐55.9)	88.9 (65.3‐98.6)	38.9 (27.6‐51.1)	90.0 (55.5‐99.8)	36.3 (25.8‐47.8)
SDMA > 14 μg/dL	90	74.2 (55.4‐88.1)	55.9 (42.4‐68.8)	72.2 (46.5‐90.3)	50.0 (38.0‐62.0)	90.0 (55.5‐99.8)	50.0 (38.6‐61.4)
SDMA > 16 μg/dL	90	62.5 (43.7‐78.9)	79.3 (66.7‐88.8)	66.7 (41.0‐86.7)	72.2 (60.4‐82.1)	90.0 (55.5‐99.8)	71.3 (60.1‐80.8)
SDMA > 18 μg/dL	90	45.2 (27.3‐64.0)	94.9 (85.9‐98.9)	55.6 (30.8‐78.5)	90.3 (81.0‐96.0)	80.0 (44.4‐97.5)	88.8 (79.7‐94.7)
SDMA > 20 μg/dL	90	29.0 (14.2‐48.0)	94.9 (85.9–98.9)	44.4 (21.5‐69.2)	94.4 (86.4‐98.5)	60.0 (26.2‐87.8)	92.5 (84.4‐97.2)
Creatinine ≥ 1.0 mg/dL	90	87.1 (70.2‐96.4)	52.5 (39.1‐65.7)	88.9 (65.3‐98.6)	45.8 (34.0‐58.0)	90.0 (55.5‐99.8)	42.5 (31.5‐54.1)
Creatinine ≥ 1.1 mg/dL	90	83.9 (66.3‐94.6)	67.8 (54.4‐79.4)	88.9 (65.3‐98.6)	59.7 (47.5‐71.1)	90.0 (55.5‐99.8)	55.0 (43.5‐66.2)
Creatinine ≥ 1.2 mg/dL	90	71.0 (52.0‐85.8)	81.4 (69.1‐90.3)	83.3 (58.6‐96.4)	75.0 (63.4‐84.5)	90.0 (55.5‐99.8)	70.0 (58.7‐79.7)
Creatinine ≥ 1.3 mg/dL	90	64.5 (45.4‐80.8)	88.1 (77.1‐95.1)	83.3 (58.6‐96.4)	83.3 (72.7‐91.1)	90.0 (55.5‐99.8)	77.5 (66.8‐86.1)
Creatinine ≥ 1.4 mg/dL	90	35.5 (19.2‐54.6)	96.6 (88.3‐99.6)	50.0 (26.0‐74.0)	94.4 (86.4‐98.5)	70.0 (34.8‐93.3)	92.5 (84.4‐97.2)
Creatinine ≥ 1.5 mg/dL	90	9.7 (2.0‐25.8)	100.0 (93.9‐100.0)	5.6 (0.1‐27.3)	97.2 (90.3‐99.7)	10.0 (0.3‐44.5)	97.5 (91.3‐99.7)

Abbreviations: GFR, glomerular filtration rate; SDMA, symmetric dimethylarginine.

**Table 3:** Positive likelihood ratios (LR+) and negative likelihood ratios (LR−) of different cutoff points for SDMA and serum creatinine concentration to detect different categories of %GFR decrease below the mean GFR of the patient's body weight category

Variables	Sample size	LR+ %GFR ↓ ≥20% (95% CI)	LR− %GFR ↓ ≥20% (95% CI)	LR+ %GFR ↓ ≥30% (95% CI)	LR− %GFR ↓ ≥30% (95% CI)	LR+ %GFR ↓ ≥40% (95% CI)	LR− %GFR ↓ ≥40% (95% CI)
SDMA > 10 μg/dL	90	1.2 (1.1‐1.4)	0.2 (0.0‐1.1)	1.2 (1.0‐1.4)	0.3 (0.0‐2.2)	1.1 (0.9‐1.3)	0.6 (0.1‐3.8)
SDMA > 12 μg/dL	90	1.5 (1.1‐1.9)	0.4 (0.2‐0.9)	1.5 (1.1‐1.9)	0.3 (0.1‐1.1)	1.4 (1.1‐1.8)	0.3 (0.0‐1.8)
SDMA > 14 μg/dL	90	1.7 (1.2‐2.4)	0.5 (0.2‐0.9)	1.4 (1.0‐2.1)	0.6 (0.3‐1.2)	1.8 (1.3‐2.4)	0.2 (0.0‐1.3)
SDMA > 16 μg/dL	90	3.0 (1.7‐5.1)	0.5 (0.3‐0.8)	2.4 (1.5‐3.9)	0.5 (0.2‐0.9)	3.1 (2.1‐4.7)	0.1 (0.0‐0.9)
SDMA > 18 μg/dL	90	8.9 (2.8‐28.6)	0.6 (0.4‐0.8)	5.7 (2.5‐12.9)	0.5 (0.3‐0.8)	7.1 (3.6‐14.2)	0.2 (0.1‐0.8)
SDMA > 20 μg/dL	90	5.7 (1.7‐19.6)	0.7 (0.6‐0.9)	7.9 (2.7‐23.6)	0.6 (0.4‐0.9)	8.0 (3.2‐20.1)	0.4 (0.2‐0.9)
Creatinine ≥ 1.0 mg/dL	90	1.8 (1.4‐2.5)	0.3 (0.1‐0.6)	1.6 (1.3‐2.1)	0.2 (0.1‐0.9)	1.6 (1.2‐2.1)	0.2 (0.0‐1.5)
Creatinine ≥ 1.1 mg/dL	90	2.6 (1.7‐3.9)	0.2 (0.1‐0.5)	2.2 (1.6‐3.1)	0.2 (0.1‐0.7)	2.0 (1.5‐2.8)	0.2 (0.0‐1.2)
Creatinine ≥ 1.2 mg/dL	90	3.8 (2.1‐6.8)	0.4 (0.2‐0.6)	3.3 (2.1‐5.2)	0.2 (0.1‐0.6)	3.0 (2.0‐4.5)	0.1 (0.0‐0.9)
Creatinine ≥ 1.3 mg/dL	90	5.4 (2.6‐11.4)	0.4 (0.3‐0.7)	5.0 (2.9‐8.7)	0.2 (0.1‐0.6)	4.0 (2.5‐6.3)	0.1 (0.0‐0.8)
Creatinine ≥ 1.4 mg/dL	90	10.4 (2.5‐44.3)	0.7 (0.5‐0.9)	8.9 (3.1‐25.9)	0.5 (0.3‐0.8)	9.3 (3.9‐22.3)	0.3 (0.1‐0.8)
Creatinine ≥ 1.5 mg/dL	90	—	0.9 (0.8‐1.0)	2.0 (0.2‐20.9)	1.0 (0.9‐1.1)	4.0 (0.4‐40.3)	0.9 (0.8‐1.1)

*Notes*: The LR+ is the probability of a dog with decreased GFR to have the analyte exceeding cutoff divided by the probability of a dog with normal GFR to have the same analyte exceeding cutoff ($\mathrm{LR}+=\frac{\text{sensitivity}\;}{1-\text{specificity}})$. A LR+ greater than 1 indicates that the test result is associated with decreased GFR. The higher the LR+, the stronger the association. The LR− is the probability of a dog with decreased GFR to have the analyte below the cutoff divided by the probability of a dog with normal GFR to have the same analyte below the cutoff ($\mathrm{LR}-=\frac{1-\text{sensitivity}\;}{\text{specificity}})$. A likelihood ratio less than 1 indicates that the result is associated with absence of the disease. The lower the LR− (trending towards 0), the stronger the exclusion.

Abbreviations: GFR, glomerular filtration rate; SDMA, symmetric dimethylarginine.

The LR+ is the probability of a dog with decreased GFR to have the analyte exceeding cutoff divided by the probability of a dog with normal GFR to have the same analyte exceeding cutoff ($\mathrm{LR}+=\frac{\text{sensitivity}\;}{1-\text{specificity}})$. A LR+ greater than 1 indicates that the test result is associated with decreased GFR. The higher the LR+, the stronger the association. The LR− is the probability of a dog with decreased GFR to have the analyte below the cutoff divided by the probability of a dog with normal GFR to have the same analyte below the cutoff ($\mathrm{LR}-=\frac{1-\text{sensitivity}\;}{\text{specificity}})$. A likelihood ratio less than 1 indicates that the result is associated with absence of the disease. The lower the LR−, the stronger the exclusion.

**Figure 4:**
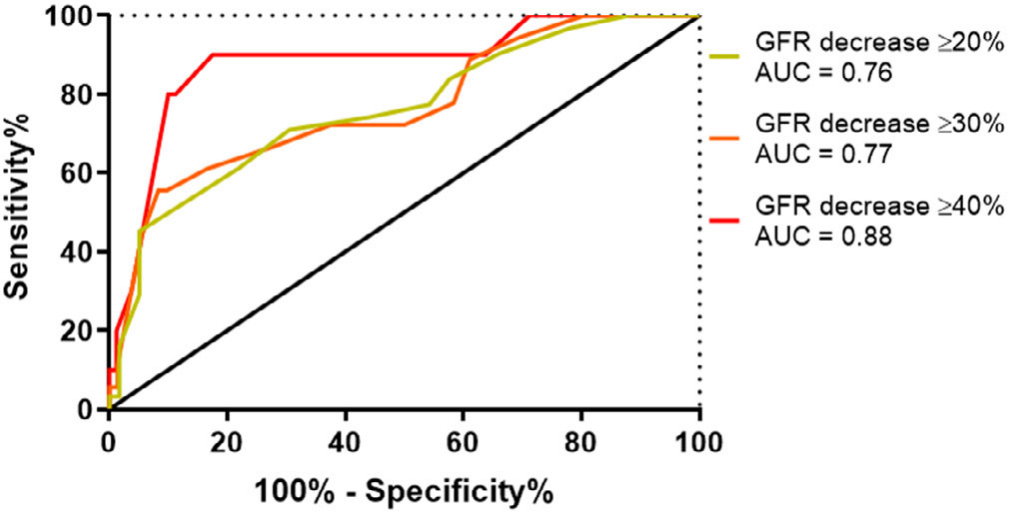
Receiver operating characteristic curve for serum symmetric dimethylarginine detecting a glomerular filtration rate (GFR) decrease of ≥20%, ≥30%, and ≥40% below the mean GFR of a dog's body weight category. AUC, area under the curve

A ROC curve assessing the trade‐off between sensitivity and (1 − specificity) across the range of SDMA concentrations (including the cutoff points of >10, >12, >14, >16, >18, and >20 μg/dL) to detect decreases in GFR below the mean GFR of a canine body weight category of ≥20%, ≥30%, and ≥40% is presented in Figure 4. The area under the curve for SDMA assessing for a decrease in GFR ≥20% was 0.76, 0.77 for assessing for a GFR decrease ≥30%, and 0.88 for assessing for a GFR decrease ≥40%. Based off ROC curve data, using Youden's J statistic, the optimal SDMA cutoff for assessing for a GFR decrease of ≥20% was >18 μg/dL (sensitivity 52% [95% CI, 33‐70], specificity 88% [95% CI, 77‐95]). The optimal SDMA cutoff for assessing for a GFR decrease of ≥30% was >20 μg/dL (sensitivity 56% [95% CI, 31‐78], specificity 92% [95% CI, 83‐97]), and optimal SDMA cutoff for assessing for a GFR decrease of ≥40% was >18 μg/dL (sensitivity 90% [95% CI, 55‐100], specificity 83% [95% CI, 72‐90]).

**Figure 5:**
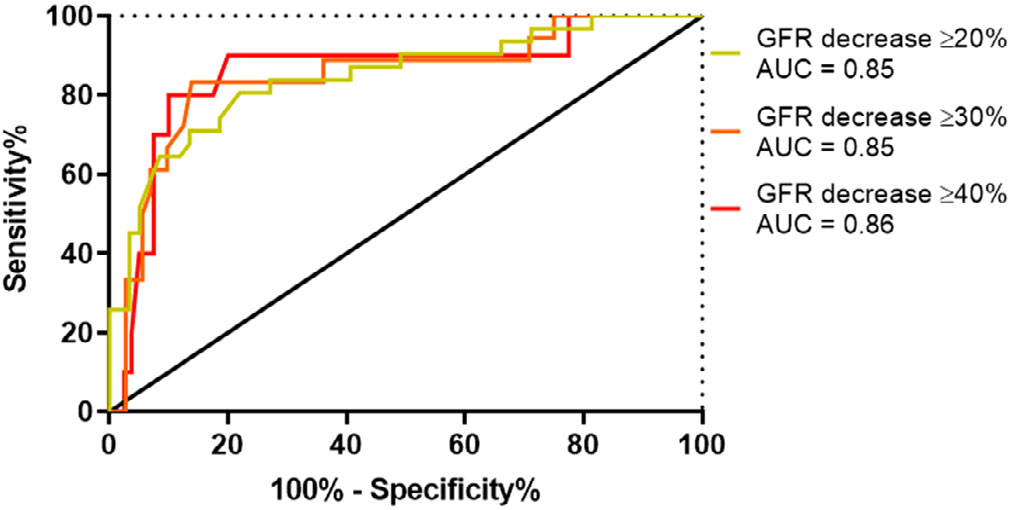
Receiver operating characteristic curve for serum creatinine detecting a glomerular filtration rate (GFR) decrease of ≥20%, ≥30%, and ≥40% below the mean GFR of a dog's body weight category. AUC, area under the curve

A ROC curve assessing the trade‐off between sensitivity and (1 − specificity) across the range of serum creatinine concentrations (including the cutoff points of ≥1.0, ≥1.1, ≥1.2, ≥1.3, ≥1.4, and ≥1.5 mg/dL) to detect decreases in GFR below the mean GFR of a canine body weight category of ≥20%, ≥30% and ≥40% is presented in Figure 5. The area under the curve for serum creatinine assessing for a decrease in GFR ≥20% was 0.85, 0.85 for assessing for a GFR decrease ≥30%, and 0.86 for assessing for a GFR decrease ≥40%. Based off ROC curve data, using Youden's J statistic, the optimal serum creatinine concentration cutoff for assessing for a GFR decrease of ≥20% was ≥1.14 mg/dL (sensitivity 81% [95% CI, 63‐93], specificity 76% [95% CI, 63‐86]). The optimal serum creatinine concentration for assessing for a GFR decrease of ≥30% was ≥1.31 mg/dL (sensitivity 83% [95% CI, 59‐96], specificity 86% [95% CI, 76‐93]) and optimal serum creatinine concentration for assessing for a GFR decrease of ≥40% was ≥1.36 mg/dL (sensitivity 80% [95% CI 44‐98], specificity 89% [95% CI 80‐95]).

Nine of 90 (10%) dogs had concurrent SDMA >18 μg/dL and serum creatinine ≥1.36 mg/dL documented. Manually calculated sensitivity and specificity of concurrent SDMA >18 μg/dL and serum creatinine ≥1.36 mg/dL for detection of a GFR decrease of ≥40% below mean were 70% (95% CI, 35‐93) and 98% (95% CI, 91‐100), respectively. Manually calculated LR+ and LR− of concurrent SDMA >18 μg/dL and serum creatinine ≥1.36 mg/dL for detection of a GFR decrease of ≥40% below mean were 28.0 (95% CI, 6.7‐116.7) and 0.3 (95% CI, 0.1‐0.8) respectively.

Out of the 59 dogs that had increased SDMA (>14 μg/dL) measured, in 32 dogs (54%) their GFR result was not considered consistent with renal disease (GFR increased or <20% decreased from the mean GFR of their body weight category). Out of these 32 dogs, reviewing the available follow‐up data, a final diagnosis was available for 23 (71.8%), with a median time to follow‐up of 344 days (range, 2‐951 days). A range of different final diagnoses for the dogs' presenting clinical signs were obtained: psychogenic polydipsia (n = 8), idiopathic dermatopathy (n = 5), clinically normal (n = 3), central diabetes insipidus (n = 2), colonic adenocarcinoma (n = 1), urinary tract infection (n = 1), urinary incontinence (n = 1), pyelonephritis (n = 1), and renal mass (n = 1). Follow‐up data on dogs that had a decrease in GFR ≥20% below mean is further discussed in a separate publication.^23^

## 4 DISCUSSION

The relationships between GFR and both serum creatinine and SDMA were linear. This contrasts with data from a previous study,^19^ where the relationships between GFR and both markers were nonlinear. A likely explanation for this different relationship is that the population of dogs in our study either had normal or mildly reduced kidney function, resulting in a limited range of GFR results, whereas the population of dogs in the earlier study included dogs that were azotemic.^19^ The correlations between SDMA and GFR and between serum creatinine and GFR in this study were lower than previously reported.^19^ Again, a likely explanation for this is the limited range of GFR results in our population. Another possible explanation is that the dogs in our study represented a heterogeneous population of client‐owned dogs, which were ultimately diagnosed with a variety of medical conditions that could have influenced serum creatinine or SDMA concentrations.

Increased SDMA above the reference interval of 14 μg/dL was sensitive (90%) but nonspecific (50%) for detection of a ≥40% GFR decrease below the mean GFR of the canine body weight category. This percentage decrease in GFR is considered a clear indication of a renal etiology of disease. Using ROC curve analysis and Youden's J statistic, the optimal SDMA cutoff for assessing for a GFR decrease of ≥40% was 18 μg/dL (sensitivity 90%, specificity 83%). Given the low specificity of SDMA >14 μg/dL for detecting a GFR decrease of ≥40%, the authors suggest that applying a cutoff of >18 μg/dL could be more appropriate than the previously described reference interval when used to test for decreased renal function in dogs with a clinical presentation suggestive of non‐azotemic renal disease as a cause for polyuria and polydipsia or other urinary tract signs. When using SDMA to screen for non‐azotemic renal disease, if using the traditional reference interval of >14 μg/dL, only half (50%) of the cases will ultimately have a GFR decrease of ≥40% below the mean GFR for the canine body weight category. The specificity is greatly improved (83%), while maintaining the same sensitivity (90%), if the SDMA cutoff is changed to >18 μg/dL even if serum creatinine is within the reference interval.

In 54% of dogs who had SDMA measured above reference interval (>14 μg/dL), GFR was increased or <20% decreased below the mean GFR of the canine body weight category. The fact that more than half of the dogs with increased SDMA did not have a GFR result consistent with decreased renal function raises concern for false‐positive results in this scenario had SDMA alone been used (as a single data point) to interpret renal function in dogs being screened for non‐azotemic renal disease. Evaluating the final diagnosis from follow‐up clinical data for dogs with increased SDMA but normal GFR estimation results, several dogs fell into the category of idiopathic dermatopathy. Many of these cases had GFR estimation performed to screen for AKI due to the presence of skin lesions, reflecting the recent emergence of cutaneous and renal glomerular vasculopathy in the United Kingdom.^26^ Three dogs with increased SDMA and an increase or ≤20% decrease from the mean GFR of their body weight category were considered clinically normal by the submitting veterinarians at the time of follow‐up; unfortunately, this assessment was based on spontaneous resolution of clinical signs (polyuria‐polydipsia in all cases) rather than on longitudinal monitoring of their kidney function. Therefore, the authors cannot exclude the possibility that these dogs could have had undetected kidney disease at the time of follow‐up and that progression of kidney disease would have been documented either through serial assessment of serum creatinine concentration or repeat GFR estimation. The ultimate classification of these dogs as normal can therefore be questioned. Serial assessment of SDMA and GFR estimation in these dogs would have been of interest to determine the long‐term outcome of carefully monitored renal function in these cases with initially increased SDMA concentrations. However, this was unfortunately not possible within the scope of this study.

A common rationale for measuring SMDA or performing GFR estimation in dogs is to determine whether a renal etiology exists for presenting clinical signs, typically polyuria and polydipsia, in non‐azotemic patients. All dogs included in the statistical analysis for this study were non‐azotemic at the time of GFR estimation. With ROC curve analysis and Youden's J statistic, the optimal serum creatinine cutoff for assessing for a GFR decrease of ≥40% was ≥1.36 mg/dL (sensitivity 80%, specificity 89%). This cutoff for serum creatinine had lower sensitivity (80% vs. 90%) but greater specificity (89% vs. 83%) to using a cut‐off of >18 mg/dL for SMDA for detection of a GFR decrease of ≥40%. The presence of a concurrent serum creatinine concentration of ≥1.36 mg/dL in conjunction with an SDMA result of >18 mg/dL had greater specificity (98%) but lower sensitivity (70%) for detecting a GFR decrease of ≥40% than documentation of either serum creatinine ≥1.36 mg/dL or SDMA >18 mg/dL alone.

The authors acknowledge that using breed‐specific reference intervals for serum creatinine or serial monitoring of serum creatinine concentrations may have increased the sensitivity of creatinine for detection of decreased renal function without the requirement for GFR estimation in some of the dogs in the present study. However, breed‐specific reference intervals and serial creatinine concentrations from the same laboratory were not available, so it was not possible to compare the sensitivity of SMDA to the use of breed‐specific creatinine reference intervals or to serial creatinine measurements. Such a comparison could be a focus for a future study.

The authors recognize the limitations of this study. Firstly, this study relied on submitting veterinarians following a standard iohexol clearance protocol. To the best of the authors' knowledge, the recommended protocol was followed by submitting veterinarians, but we acknowledge the possibility that in some cases, deviation from the protocol could have occurred with subsequent effects on the results. The canine‐specific correction formula used to calculate estimated GFR in this study was derived from data from dogs undergoing surgery for pyometra,^27^ whereas our population was much more heterogeneous. As the correction formula has not been validated in a population as varied as in this study, it is possible that the extrapolation of this correction formula to a heterogeneous population could have decreased the accuracy of GFR estimation calculations. Symmetric dimethylarginine samples in the study were submitted to 2 different laboratories: Idexx Wetherby (UK, n = 96) and Idexx Ludwigsburg (Germany, n = 17). The reference intervals, analyzers, and methodology used to measure SDMA were identical in both laboratories; as such, the authors acknowledge that the fact that SDMA was measured at 2 different laboratories, albeit from the same company, could have affected some of the results in this study, but believe it is unlikely that this impacts the validity of the results. A further limitation relates to the storage times of serum samples for serum creatinine and SDMA measurement. Although the median time these serum samples were stored in a −80°C freezer was 166 days, some samples were stored for a much longer time (up to 1360 days) which could have impacted results if sample preservation was compromised.

The authors also acknowledge the limitations of using GFR estimation as the gold standard for assessment of renal function. Glomerular filtration rate varies with the size of the dog (somewhat accounted for by body weight)^12^ and with breed,^28^ which could lead to misinterpretation of renal function of a dog if its size and breed is not taken into account. It is also possible that comparing a dog's GFR to the mean GFR of the dog's body weight category^13^ may lead to misinterpretation of renal function of a dog; the mean GFR of their body weight category may not reflect what is a normal GFR for an individual dog, which could lead to overestimation or underestimation of any reduction in GFR. Information on body condition score and muscle condition score was not available for the dogs in our study population. As actual, rather than ideal, body weight was used to classify dogs into weight quartiles, it is possible that some dogs could have been misclassified into a higher or lower body weight quartile, potentially leading to comparison of their GFR value to an inappropriate mean. In addition, 1 dog's body weight did not fall within the ranges of the defined body weight categories,^13^ and therefore this dog was included in the body weight category to which its body weight was closest. The authors acknowledge the possibility that this dog could have been misclassified and its GFR compared to an inappropriate mean.

In conclusion, SDMA can be a sensitive marker for detecting decreased renal function in non‐azotemic dogs depending on the cutoff used. In non‐azotemic dogs being screened for decreased renal function, defined as a GFR decrease ≥40% below the mean GFR of their body weight category, using a cutoff of >18 μg/dL rather than the traditional cutoff of >14 μg/dL increases the specificity of SDMA, without compromising sensitivity in this population.

## CONFLICT OF INTEREST DECLARATION

Ludovic Pelligand has affiliation with deltaDOT through a Concept Development Partnership (shared company/RVC investment) which resulted in employment of a postdoctoral researcher for 4 years for the development of the GFR service.

## OFF‐LABEL ANTIMICROBIAL DECLARATION

Authors declare no off‐label use of antimicrobials.

## INSTITUTIONAL ANIMAL CARE AND USE COMMITTEE (IACUC) OR OTHER APPROVAL DECLARATION

This project was reviewed and approved by the Royal Veterinary College (RVC) Social Sciences Research Ethical Review Board (reference URN SR2017‐1223) who granted approval for access to joint iohexol clearance test submission forms held by deltaDOT Ltd. and the RVC and for contact with the veterinarians for access to the clinical records of the dogs under investigation and for completion of a short questionnaire regarding case outcomes. Subsequent submission of serum samples for SDMA and serum creatinine measurement via batch analysis to Idexx Laboratories Ltd. was approved by the Royal Veterinary College (RVC) clinical research and ethical review board (reference URN 2016‐1553).

## HUMAN ETHICS APPROVAL DECLARATION

Authors declare human ethics approval was not needed for this study.
